# Assessing the Efficacy of Acanthoic Acid Isolated from *Acanthopanax koreanum* Nakai in Male Infertility: An In Vivo and In Silico Approach

**DOI:** 10.3390/cimb46070440

**Published:** 2024-07-13

**Authors:** Nguyen Viet Phong, Hyo-Sung Kim, Hyun-Jung Park, Eunbyul Yeom, Seo Young Yang

**Affiliations:** 1 Department of Biology Education, Teachers College and Institute for Phylogenomics and Evolution, Kyungpook National University, Daegu 41566, Republic of Korea; ngvietphong@gmail.com; 2 School of Life Science and Biotechnology, College of Natural Sciences, Kyungpook National University, Daegu 41566, Republic of Korea; gytjd98@gmail.com; 3 School of Life Sciences, BK21 FOUR KNU Creative BioResearch Group, Kyungpook National University, Daegu 41566, Republic of Korea; 4 KNU-G LAMP Project Group, KNU-Institute of Basic Sciences, School of Life Sciences, College of Natural Sciences, Kyungpook National University, Daegu 41566, Republic of Korea; 5 Department of Plant Life and Resource Science, Sangji University, Wonju-si 26339, Republic of Korea; parkhj02@sangji.ac.kr

**Keywords:** *Acanthopanax koreanum*, acanthoic acid, male infertility, egg-hatching rates, molecular docking, molecular dynamics

## Abstract

Acanthoic acid, a diterpene isolated from the root bark of *Acanthopanax koreanum* Nakai, possesses diverse pharmacological activities, including anti-inflammatory, anti-diabetic, gastrointestinal protection, and cardiovascular protection. This study is the first to investigate the egg-hatching rates of *Drosophila melanogaster* affected by acanthoic acid. Notably, male flies supplemented with 10 μM acanthoic acid exhibited a strong increase in hatching rates compared with controls under adverse temperature conditions, suggesting a potential protective effect against environmental stressors. Molecular docking simulations revealed the binding affinities and specific interactions between acanthoic acid and proteins related to male infertility, including SHBG, ADAM17, and DNase I, with binding affinity values of −10.2, −6.8, and −5.8 kcal/mol, respectively. Following the docking studies, molecular dynamic simulations were conducted for a duration of 100 ns to examine the stability of these interactions. Additionally, a total binding energy analysis and decomposition analysis offered insights into the underlying energetic components and identified key contributing residues.

## 1. Introduction

Male infertility, affecting approximately 7% of males worldwide, stems from a variety of factors, including genetic mutations, hormonal imbalances, and lifestyle influences [1]. Genetic anomalies, such as structural abnormalities in the X and Y chromosomes, can disrupt spermatogenesis, reducing sperm count and quality [2]. Environmental factors like radiation, drug overdose, and pollutants further exacerbate these issues by impacting the hypothalamic–pituitary–gonadal axis, which controls sperm production [3,4]. With the 50% to 60% decline in sperm counts observed in several regions between 1973 and 2011, the need for effective treatments is more urgent than ever [5].

Natural products offer a promising alternative to treat male infertility. Herbal extracts such as ginseng (*Panax ginseng*), *Tribulus terrestris*, and maca (*Lepidium meyenii*) are known to boost testosterone levels and improve sperm count and motility [6,7,8]. Secondary metabolites from medicinal plants are also gaining attention for their potential benefits for male fertility. Epigallocatechin gallate, found in green tea, has been shown to enhance sperm motility and reduce DNA fragmentation [9]. Resveratrol, a compound found in grapes and red wine, has antioxidant properties that protect sperm from oxidative damage [10]. Quercetin, commonly derived from apples and onions, has demonstrated anti-inflammatory effects that may support spermatogenesis [11]. These natural solutions offer a safer and less invasive approach to treating male infertility, complementing surgical and assisted reproductive technologies.

*Acanthopanax koreanum* Nakai, belonging to the Araliaceae family, was first discovered on Jeju Island in South Korea and is recognized as a distinct species within the Acanthopanax genus [12]. Traditionally, *A. koreanum* has been used as a tonic agent [13] and has been valued for its effectiveness in treating conditions such as joint and bone pain, rheumatism, cardiovascular disease, diabetes, and hepatitis [12,14,15]. Among the secondary metabolites isolated from *A. koreanum*, acanthoic acid, a pimarane-type diterpene, has demonstrated various pharmacological benefits, including anti-cancer, anti-inflammatory, anti-diabetes, antimicrobial, liver protection, and gastrointestinal protection effects [16,17,18,19]. Furthermore, acanthoic acid is an agonist of liver X receptors and is crucial in managing hepatic fibrosis, lipid metabolism, cardiovascular health, and inflammation [20,21,22]. The potent anti-inflammatory effect of acanthoic acid is a key reason for its therapeutic benefits, as it helps to suppress the release of pro-inflammatory factors and blocks the activation of the toll-like receptor 4/nuclear factor-kappa B pathway [20,21].

To continue research into the potential effects of acanthoic acid, this study investigated the efficacy of acanthoic acid for male infertility using a *Drosophila* egg-hatching test for the first time. In addition, in silico studies, including molecular docking and molecular dynamics (MD) simulations, were conducted to further investigate the binding affinity, interactions, and conformational stability of acanthoic acid with proteins related to male infertility.

## 2. Materials and Methods

### 2.1. Plant Material

*A. koreanum* root bark was purchased from a traditional market in Jeju, Republic of Korea, in September 2023. The plant was authenticated by one of the authors, Professor Seo Young Yang. A specimen voucher of this plant (KNU-2023AK) was deposited at the Institute for Phylogenomics and Evolution, Kyungpook National University.

### 2.2. Isolation of Acanthoic Acid

Acanthoic acid was isolated using a previously established procedure [14]. Briefly, dried root bark from *A. koreanum* (2 kg) was extracted using methanol (MeOH) under reflux conditions three times, yielding 192 g of MeOH extract. This extract was suspended in water and partitioned using CH_2_Cl_2_. The CH_2_Cl_2_ fraction was concentrated under reduced pressure (in vacuo), resulting in 96 g of CH_2_Cl_2_ extract. The CH_2_Cl_2_ extract was subjected to silica gel column chromatography and eluted using a gradient mixture of *n*-hexane and EtOAc (20:1–1:1, *v*/*v*). It was further purified using Sephadex LH-20 column chromatography with a mixture of MeOH and H_2_O (4:1, *v*/*v*) as the eluent, leading to the isolation of acanthoic acid. The chemical structure of acanthoic acid was confirmed by NMR spectroscopy.

### 2.3. Drosophila Egg-Hatching Rate Analysis

To assess the effects of temperature on the reproductive capacity of *Drosophila melanogaster*, eggs were collected from a freshly prepared vial containing a food–compound mixture over a six-hour period. These vials were then placed in an incubator maintained at 28 °C, allowing the eggs to develop into adult flies. Male flies from this cohort were mated with virgin females that were raised at 25 °C without exposure to any compound. Post-mating, the flies were transferred to grape agar plates to lay eggs overnight. The number of deposited eggs was counted the following day. To evaluate fertility, the grape agar plates were incubated at 25 °C, and egg hatching was monitored for 48 h. The number of hatched eggs and the hatching rate were quantified and recorded.

### 2.4. Molecular Docking

Molecular docking simulations were conducted to investigate the binding and interaction of acanthoic acid with proteins implicated in male infertility. The simulations were performed using AutoDock Vina 1.1.2 [23] based on an established procedure [24]. Crystallographic structures of human sex hormone-binding globulin (SHBG; PDB ID: 1KDM) [25], a disintegrin and metalloproteinase 17 (ADAM17; PDB ID: 2I47) [26], and deoxyribonuclease I (DNase I; PBD ID: 4AWN) [27] were retrieved from the Protein Data Bank to set up the simulations. The protein structures were prepared using BIOVIA Discovery Studio Visualizer v21 (Dassault Systèmes, Waltham, MA, USA), while the 3D structure of acanthoic acid was generated and minimized using Spartan’24 (Wavefunction, Irvine, CA, USA). These structures were then converted into the pdbqt format using Open Babel 3.1.1 [28]. Blind docking was employed to identify potential binding sites on the proteins. The docking simulations were validated in a redocking study, following a protocol described by Lima et al. [29]. RMSD values of all complexes of acanthoic acid with target proteins ≤ 2 Å indicated the validation of redocking. Once the docking simulations were complete, the Discovery Studio Visualizer and the Protein–Ligand Interaction Profiler (PLIP) web tool (https://plip-tool.biotec.tu-dresden.de/plip-web/plip/index, accessed on 29 June 2024) [30] were used to visualize the binding interactions.

### 2.5. MD Simulations

MD simulations were carried out to evaluate the binding stability of the docking complexes using GROMACS 2022.1 [31,32], following our previously established protocols [33,34]. The system setup was facilitated using the CHARMM-GUI web platform with the CHARMM36 force field. Periodic boundary conditions were applied, with an explicit solvation model using TIP3P water molecules to create a cubic box. Sodium (Na^+^) and/or chloride (Cl^−^) ions were added to the system to ensure electrical neutrality and stability. The initial energy minimization of the complexes was performed, aiming for a maximum force threshold of 10 kJ/mol. Once minimized, the system underwent NVT equilibration at a temperature of 300 K and NPT ensemble relaxation at a pressure of 1.013 bar. The MD simulations were run for a total of 100 ns. Additionally, MD simulations of apo and holo complexes were performed for comparison with the complexes of acanthoic acid with target proteins.

After the simulations, the trajectory data were analyzed and visualized using PyMOL 2.5.4 (Schrödinger, New York, NY, USA) and Grace (Grace Development Team, https://plasma-gate.weizmann.ac.il/Grace/, accessed on 3 July 2024). Furthermore, hydrogen bond analysis was conducted using the GROMACS H-bond (*gmx_hbond*) module [35] and the HbMap2Grace program (https://github.com/LMDM/hbmap2grace/tree/main/, accessed on 3 July 2024) [36], and the molecular surface area analysis was conducted using the SurfinMD version 1.06p4 program (https://github.com/LMDM/surfinmd/, accessed on 3 July 2024) [37] according to a protocol described by Silva et al. [38].

### 2.6. Calculation of Interaction Energies

Following the MD simulation, a decomposition analysis was performed to determine the binding energies of amino acids within 0.1 nm of the ligand using the *gmx_MMPBSA v1.6.3* package with the molecular mechanics generalized Born surface area (MM-GBSA) method. The interaction energies were calculated based on multiple components representing different molecular forces.

GGAS represents the gas-phase interaction energies with two key components: van der Waals interaction energy (VDWAALS) and electrostatic interaction energy (EFL).

GSOLV represents the solvation-related interaction energies with two key components: solvation energy calculated using the generalized Born model (EGB) and surface area-dependent solvation energy (ESURF).

Additionally, to assess the overall energy stability of the system, the total interaction energy was calculated using the following equation: TOTAL = GGAS + GSOLV. For each component, the default settings were used, except for the dielectric constant parameters (the internal was set to 1.0, and the external was set to 80.0).

## 3. Results

### 3.1. Drosophila Egg-Hatching Rate with Acanthoic Acid

**Figure 1:**
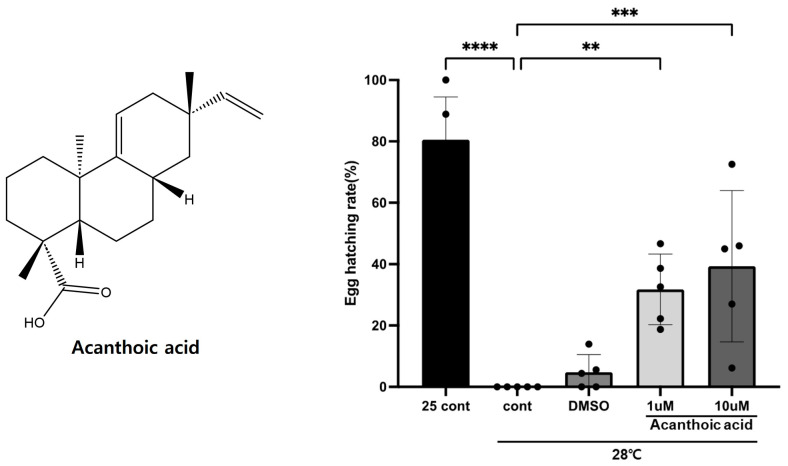
Chemical structure of acanthoic acid and the effect of acanthoic acid on *Drosophila* egg-hatching rates. The mean ± SEM; ** *p* < 0.05; *** *p* < 0.01; **** *p* < 0.001, followed by Tukey’s post hoc test.

The effect of acanthoic acid on egg-hatching rates in *Drosophila melanogaster* was investigated (Figure 1 and Table 1). Male flies were fed a diet supplemented with the test compounds, including 1 μM and 10 μM acanthoic acid [39,40]. The flies raised at 28 °C exhibited significantly lower hatching rates than the controls maintained at 25 °C. Interestingly, at 28 °C, flies on a diet supplemented with 1 μM acanthoic acid showed a notable increase in egg-hatching rates to 31.78%. Flies on a diet supplemented with 10 μM acanthoic acid showed an even greater increase, with egg-hatching rates reaching 39.33%, compared to the high-temperature group and controls fed only DMSO. A one-way ANOVA, followed by Tukey’s post hoc test, was conducted to analyze the average number of hatchings across the different treatment groups, revealing statistically significant differences among the candidate compounds.

### 3.2. Molecular Docking

**Figure 2:**
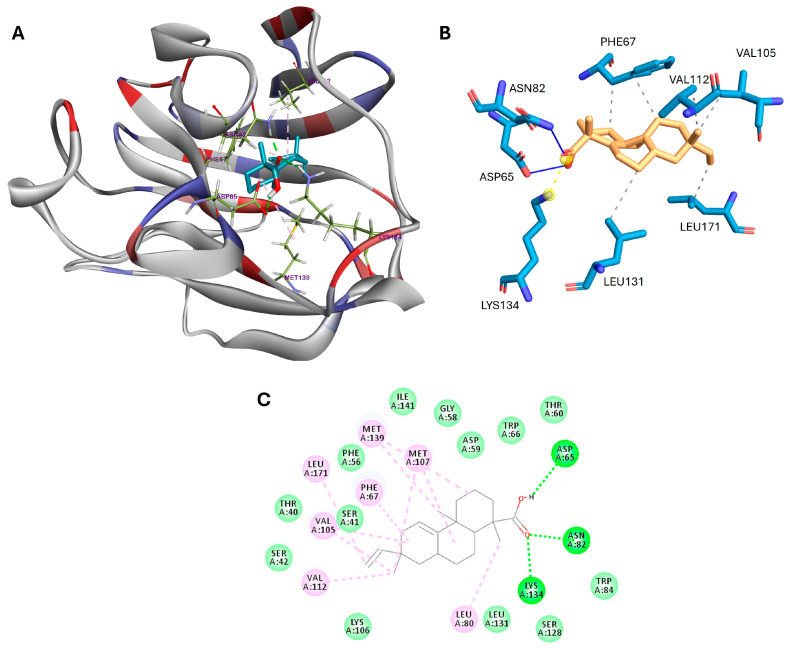
(**A**) 3D docking poses, (**B**) main interactions described by the PLIP web tool, and (**C**) 2D binding interactions for acanthoic acid with SHBG. Green: hydrogen bonds, light green: van der Waals interactions, pink: alkyl and π–alkyl interactions, and light purple: π–σ interactions.

Molecular docking simulations were conducted to assess the binding affinity and interactions of acanthoic acid with three key proteins, including SHBG, ADAM17, and DNase I, that play significant roles in male fertility. These simulations utilized blind docking and were performed using AutoDock Vina 1.1.2. As presented in Table 2, acanthoic acid interacted with SHBG, ADAM17, and DNase I with binding affinity values of −10.2, −6.8, and −5.8 kcal/mol, respectively. Human SHBG is composed of two identical monomers, each with 373 amino acids [41,42]. These monomers feature a tandem arrangement of laminin G-like domains. Only the N-terminal G domain (residues 1–194) is required for homodimer formation. As shown in Figure 2, the carboxyl group of acanthoic acid established three hydrogen bond interactions with Asp65, Asn82, and Lys134 in SHBG, while the methyl groups of acanthoic acid formed hydrophobic interactions with Leu80, Val105, Met107, Val112, and Met139.

**Figure 3:**
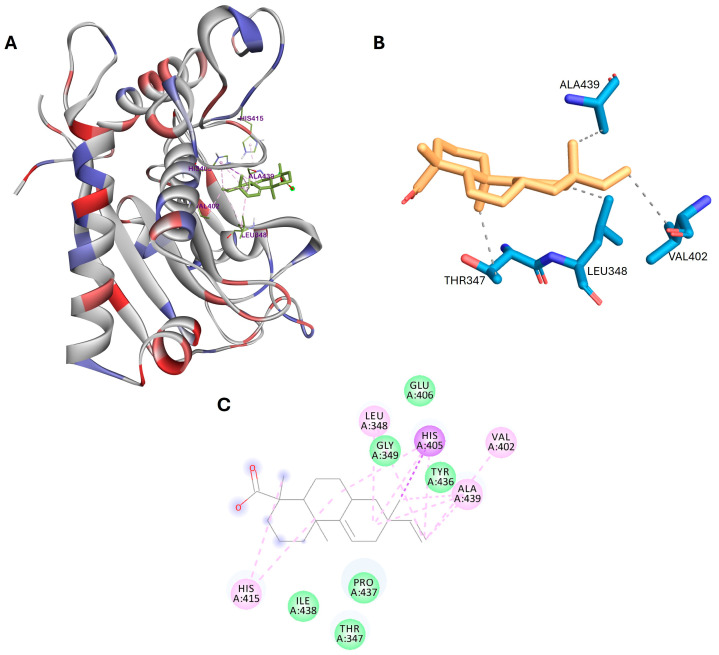
(**A**) 3D docking poses, (**B**) main interactions described by the PLIP web tool, and (**C**) 2D binding interactions for acanthoic acid with ADAM17.

Previous studies have shown that the active site of ADAM17, specifically the S1′ pocket, consists of several key amino acid residues: Leu348, Val401, Val402, His405, Val434, Ala439, and Val440 [43,44]. These residues play an important role in the ability of ADAM17 to recognize and cleave its substrates. As shown in Figure 3, acanthoic acid exhibits hydrophobic interactions with critical residues in ADAM17, including Leu348, Val402, His405, and Ala439. The other residues, Thr347, Gly349, Glu406, Tyr436, Pro437, and Ile438, interact with acanthoic acid through van der Waals forces.

**Figure 4:**
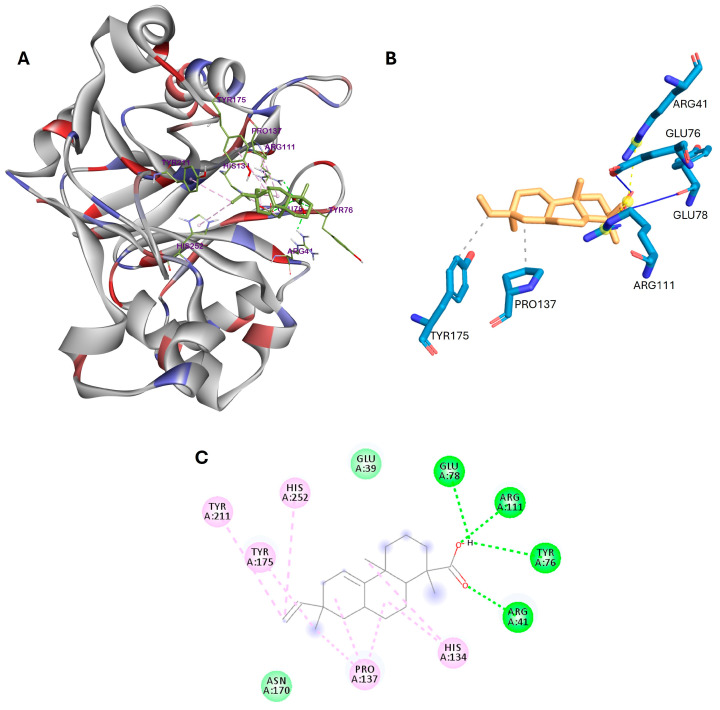
(**A**) 3D docking poses, (**B**) main interactions described by the PLIP web tool, and (**C**) 2D binding interactions for acanthoic acid with DNase I.

Furthermore, the main interactions for acanthoic acid with target proteins were visualized by the PLIP web tool [30] for each docking poses. The results are presented in Figure 2B, Figure 3B, Figure 4B, and Table S1.

In a previous study, Ulmer et al. [45] revealed that the active site of DNase I is composed of two histidine residues (His134 and His252) and two acidic residues (Glu78 and Asp212), which are essential for the acid–base catalysis of phosphodiester bonds. As shown in Figure 4, acanthoic acid can interact with two histidine residues in DNase I via π–alkyl interactions and with the acidic residue Glu78 via hydrogen bonds. The carboxyl group of this compound also forms three hydrogen bonds with Arg41, Tyr76, and Arg111. Additionally, the residues Pro137, Tyr175, and Tyr211 interact with acanthoic acid through hydrophobic interactions.

### 3.3. MD Simulations

**Figure 5:**
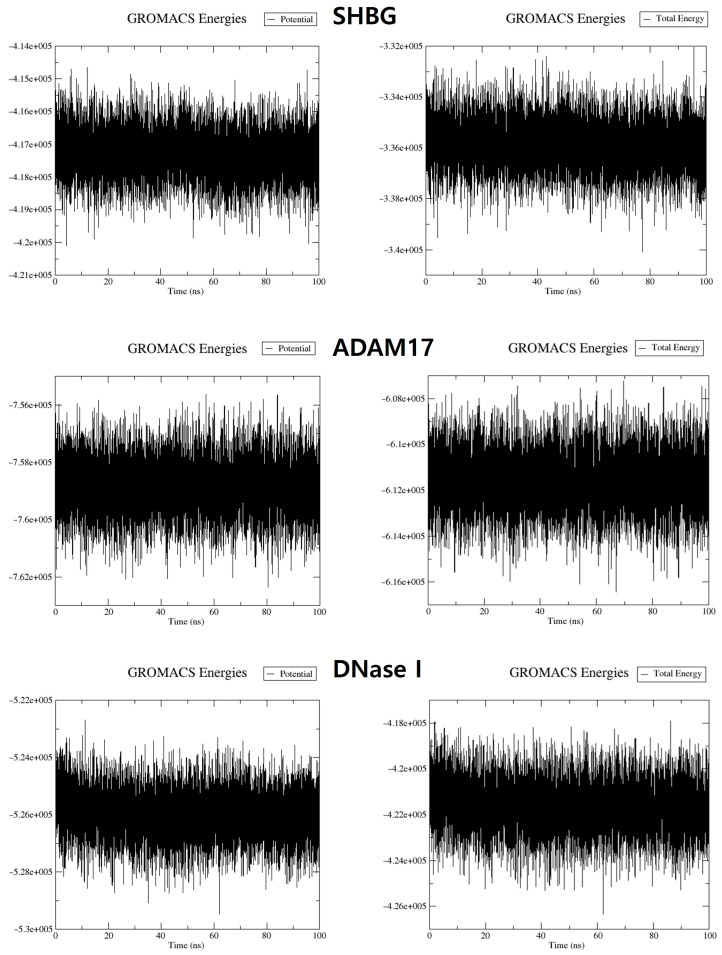
Potential and total energy values for complexes of acanthoic acid with SHBG, ADAM17, and DNase I, respectively.

To better understand the structural stability and dynamic characteristics of acanthoic acid in complexes with proteins associated with male infertility, MD simulations were performed using GROMACS 2022.1 with a duration of 100 ns. The potential energy values obtained from the MD simulations for complexes of acanthoic acid with SHBG, ADAM17, and DNase I were −416,295, −758,866, and −525,967 kJ/mol, respectively. Meanwhile, the total energy values for these complexes were −335,849, −611,581, and −421,608 kJ/mol, respectively (Figure 5).

**Figure 6:**
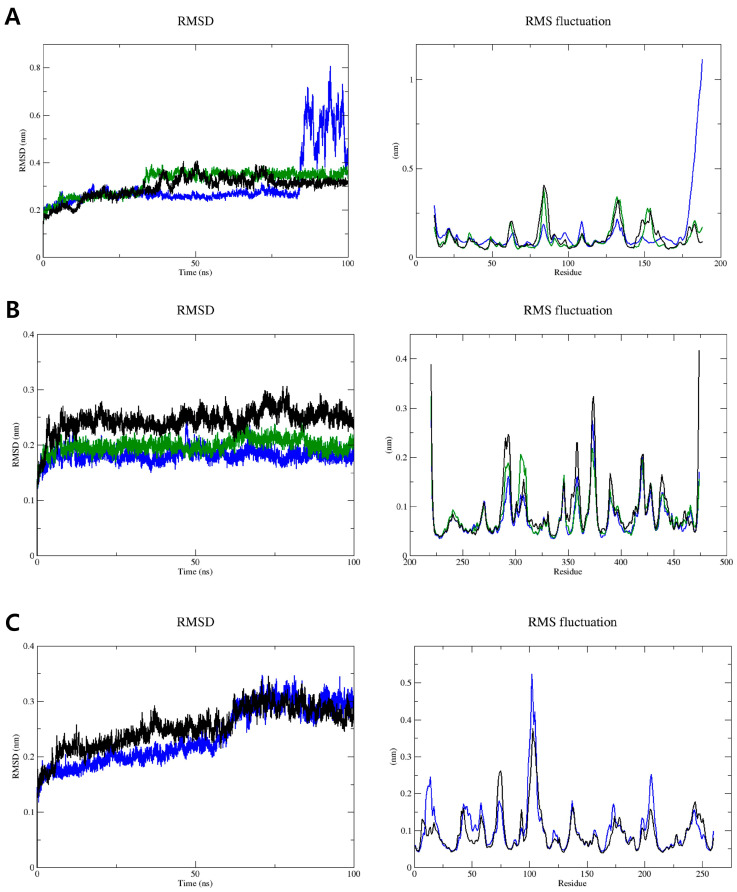
MD simulation data generated in GROMACS 2022.1 from SHBG (**A**), ADAM17 (**B**), and DNase I (**C**) complexes (apo: blue, holo: green, and acanthoic acid: black lines). RMSD (**left**) and RMSF (**right**).

Root-mean-square deviation (RMSD) analysis is a valuable method for evaluating the overall state of a simulation, providing insights into its stability over time. A lower RMSD value typically indicates that the protein–ligand complex is becoming more stable, suggesting a reduction in structural fluctuations [46]. The RMSD value for the SHBG–acanthoic acid complex gradually increased from 0.16 to 0.29 nm during the first 33 ns of the MD simulation. After this initial phase, it exhibited significant fluctuations from 0.29 to 0.40 nm between 33 and 70 ns. Afterward, the RMSD value stabilized and held steady at 0.31 nm for the remainder of the 100 ns MD trajectory, defining the productive phase from 70 to 100 ns (Figure 6A). Root-mean-square fluctuation (RMSF) analysis provides an approach to understanding the variation in individual residues within a complex throughout an MD simulation [47]. The amino acid residues Gly74–Pro76 and Pro137 exhibited notable fluctuations, with RMSF values ranging from 0.25 to 0.40 nm. Despite this flexibility, these residues are not part of the binding site where acanthoic acid interacts with SHBG, suggesting that their movement does not impact this interaction. In contrast, the individual residues within the binding site show significantly lower RMSF values consistently below 0.2 nm, indicating that the binding site of acanthoic acid with SHBG is stable.

The RMSD value for the ADAM17–acanthoic acid complex increased sharply from 0.13 to 0.23 nm during the initial 4 ns equilibration phase of the MD simulation. Subsequently, the RMSD value stabilized at approximately 0.24 nm, maintaining this level through the end of the 100 ns simulation and defining the productive phase from 45 to 100 ns (Figure 6B). Notable amino acid residues in the ADAM17 active site, such as Leu348, Val402, His405, and Ala439, showed RMSF values in the range of 0.1 to 0.15 nm, indicating the stability of the ADAM17–acanthoic acid.

Regarding DNase I inhibition, the DNase I–acanthoic acid complex exhibited an average RMSD of 0.25 nm over 100 ns of the MD simulations. Initially, the RMSD value for this complex gradually increased from 0.14 to 0.24 nm during the first 60 ns of the procedure. After a significant fluctuation at 58–60 ns, the RMSD peaked at 0.3 nm, then gradually decreased to around 0.25 nm, remaining stable at that level for the remaining simulation, defining the productive phase from 60 to 100 ns (Figure 6C). Two histidine residues, His134 and His252, and an acidic residue, Glu78, in DNase I exhibited RMSF values of 0.15 nm. Additionally, other residues in the binding site for acanthoic acid with DNase I had RMSF values below 0.2 nm, suggesting that acanthoic acid formed a stable interaction within the active site of DNase I.

The radius of gyration as a function of productive time showed that protein–acanthoic acid stabilized in all systems (Figure S1), reinforcing the RMSD data [38,48]. Furthermore, their secondary structure stability was analyzed using the DSSP (*gmx_do_dssp*) module installed on GROMACS 2022.1 [49,50]. The results indicated that all protein–acanthoic acid complexes consistently maintained a stable secondary structure during our simulation (Figure S2) [38,48].

**Figure 7:**
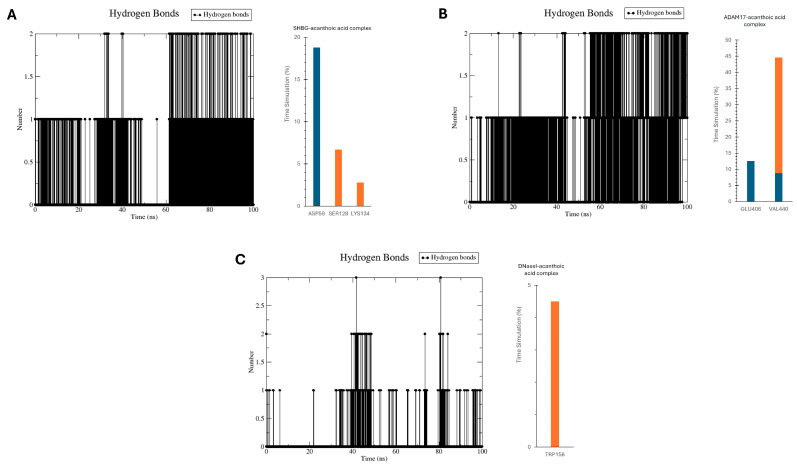
Number of hydrogen bonds and hydrogen bond stability (donor: orange and acceptor: dark blue) calculated by HbMap2Grace for the binding of acanthoic acid with SHBG (**A**), ADAM17 (**B**), and DNase I (**C**).

During the 100 ns MD simulations, acanthoic acid consistently formed 1–2 hydrogen bonds with the binding sites of SHBG and ADAM17 and 1–3 hydrogen bonds with the binding site of DNase I (Figure 7). In addition, the hydrogen bond occupancy was calculated using the H-bond (*gmx_hbond*) module and the HbMap2Grace program [36] (Figure 7). In the SHBG–acanthoic acid complex, the side chain of Asp59 acted as a hydrogen acceptor with an occupancy of 18.8%, while the side chains of Ser128 and Lys134 acted as hydrogen donors with occupancies of 6.7% and 2.8%, respectively. In the ADAM17–acanthoic acid complex, Glu406 served as a hydrogen acceptor with an occupancy of 12.6%, and Val440 functioned as both a hydrogen donor and acceptor, with occupancies of 35.8% and 8.8%, respectively. In the DNase I–acanthoic acid complex, Trp158 acted as a hydrogen donor with an occupancy of 4.5%.

**Figure 8:**
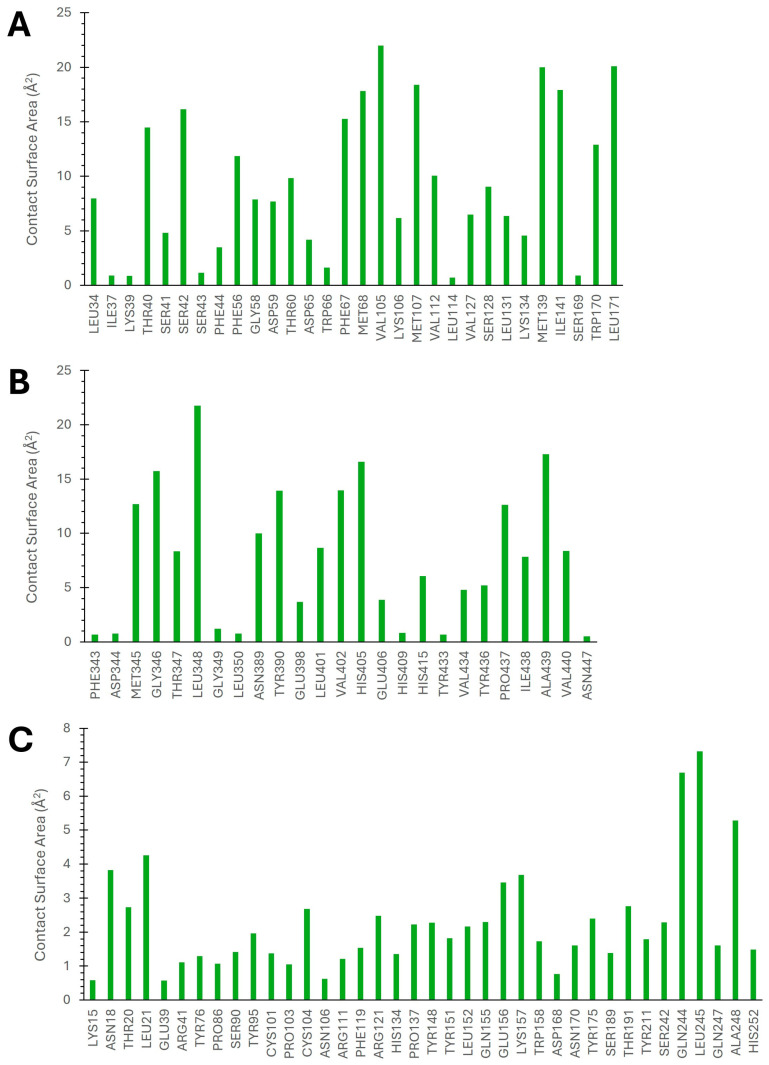
Surface molecular area (Å^2^) calculated by SurfinMD for the binding of acanthoic acid with SHBG (**A**), ADAM17 (**B**), and DNase I (**C**).

The hydrophobic interactions involving target proteins and acanthoic acid were investigated using the SurfinMD program [37] (Figure 8). In the SHBG–acanthoic acid complex, contact surface area analysis revealed interactions with residues Met68 (17.8 Å^2^), Val105 (22.0 Å^2^), Met107 (18.4 Å^2^), Met139 (20.0 Å^2^), and Leu171 (20.1 Å^2^). Additionally, interactions with Ser42 (16.1 Å^2^), Phe67 (22.0 Å^2^), and Ile141 (18.4 Å^2^) were observed. Similarly, the ADAM17–acanthoic acid complex exhibited interactions with residues Gly346 (15.7 Å^2^), Leu348 (21.8 Å^2^), Tyr390 (13.9 Å^2^), Val402 (13.9 Å^2^), His405 (16.6 Å^2^), and Ala439 (17.3 Å^2^), consistent with the findings of molecular docking studies. Contact surface area analysis of the DNase I–acanthoic acid complex revealed hydrophobic interactions beyond those identified through docking studies, involving residues Gln244 (6.7 Å^2^), Leu245 (7.3 Å^2^), and Ala248 (5.3 Å^2^) within the acanthoic acid binding site of DNase I. Overall, the contact surface area data obtained from the SurfinMD program provides strong support for the results of the molecular docking simulations.

**Figure 9:**
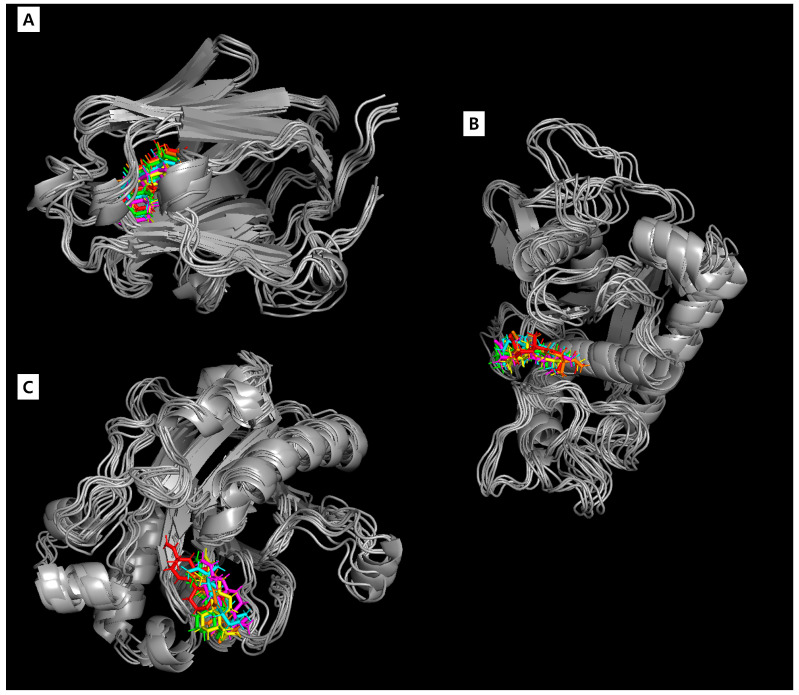
Superposition of acanthoic acid with SHBG (**A**), ADAM17 (**B**), and DNase I (**C**) during the 100 ns MD simulation. (Red: 0 ns, green: 20 ns, yellow: 40 ns, magenta: 60 ns, cyan: 80 ns, and orange: 100 ns).

The superposition of acanthoic acid with the binding sites of the target proteins was captured every 20 ns. As shown in Figure 9, no significant changes were observed in the binding site of acanthoic acid with the target proteins, suggesting the stability of the complexes involving male infertility-related proteins and acanthoic acid. Moreover, when these results were compared with the outcomes of the molecular docking studies, the MD simulation consistently displayed a similar binding mode throughout the 100 ns trajectory, validating the reliability of the computational simulation techniques in this study.

### 3.4. Total Binding Energy and Its Decomposition Analysis

**Figure 10:**
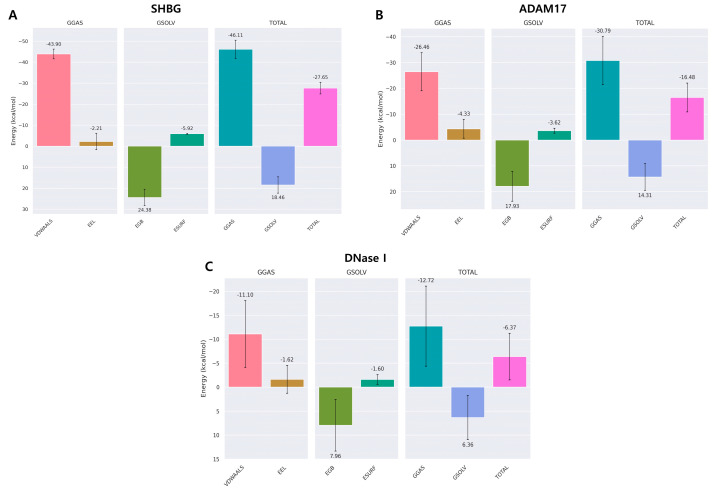
Energetic components of MM-GBSA and their average values for acanthoic acid with SHBG (**A**), ADAM17 (**B**), and DNase I (**C**). The bars represent the standard deviations for each energetic component.

The binding free energy analysis using MM-GBSA revealed several insights. As shown in Figure 10A, the total binding energy of acanthoic acid with SHBG averaged −27.65 kcal/mol. The most significant contribution came from van der Waals interactions, which averaged −43.90 kcal/mol. Electrostatic interactions averaged −2.21 kcal/mol, and the surface-area-dependent solvation energy averaged −5.92 kcal/mol.

Figure 10B depicts the energy components for acanthoic acid binding with ADAM17, showing a total energy of −16.48 kcal/mol. Here, van der Waals interactions were the most significant contributor, with an average of −26.46 kcal/mol, followed by electrostatic interactions at −4.33 kcal/mol. The total binding energy of acanthoic acid with DNase I was determined to be −6.37 kcal/mol, with van der Waals interactions averaging −11.10 kcal/mol (Figure 10C).

The correlation between the MM-GBSA energies and docking energies was investigated by comparing the MM-GBSA results with the initial docking energies for each complex (Table 3). The correlation coefficient was calculated to quantify the correlation, yielding a value of 0.96, indicating a strong positive correlation between the docking and MM-GBSA energies.

**Figure 11:**
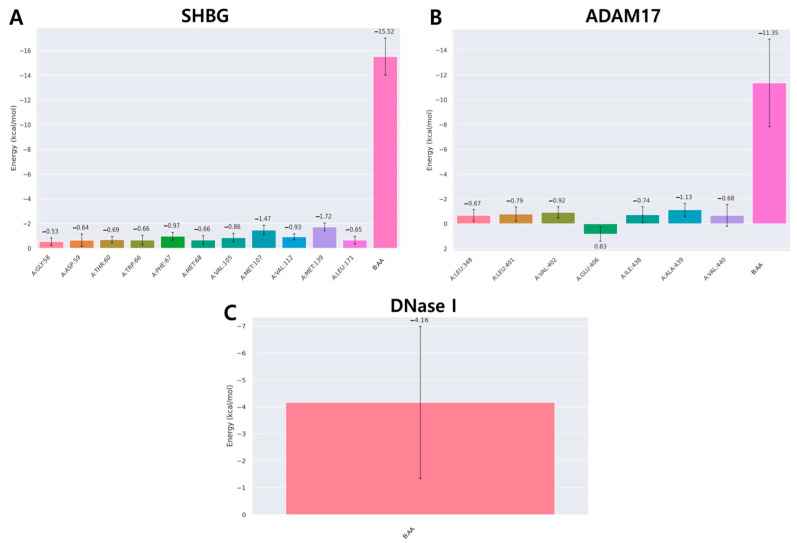
Binding free energy decomposition of the complexes of acanthoic acid with SHBG (**A**), ADAM17 (**B**), and DNase I (**C**). The figure shows the average contribution of each amino acid within 0.1 nm.

Additionally, a decomposition analysis was carried out to identify which amino acid residues within 1 nm of the ligand had the most significant contribution to the interaction (Figure 11). In the SHBG–acanthoic acid complex, two residues, Met107 (−1.47 kcal/mol) and Met139 (−1.72 kcal/mol), had contributions of less than −1 kcal/mol. In the ADAM17–acanthoic acid complex, a critical amino acid residue, Ala439, in the active site showed an energy value of −1.13 kcal/mol, the strongest contribution among all residues in this complex. Finally, no residues with contributions below −1 kcal/mol were observed in the DNase I–acanthoic acid complex.

## 4. Discussion

In this study, the impact of acanthoic acid on egg-hatching rates in *Drosophila melanogaster* under thermal stress is investigated, offering intriguing parallels to male infertility research in humans. Male infertility is a complex condition influenced by various factors, including hormonal imbalances, genetic mutations, testicular health, and heat, which can impair sperm production and quality [51]. The results indicate that male flies fed a diet containing 1 μM and 10 μM acanthoic acid exhibited significantly higher egg-hatching rates compared to controls at 28 °C, a temperature known to adversely affect fertility. Interestingly, the increase was more pronounced with higher concentrations of acanthoic acid. These findings suggest a potential role of acanthoic acid in mitigating the adverse effects of temperature on male fertility, offering promising avenues for further exploration to understand and potentially address male infertility.

Following this, molecular docking simulations were conducted to offer insights into the binding affinity and interactions of acanthoic acid with proteins related to male fertility, such as SHBG, ADAM17, and DNase I. Since the 1980s, virtual screening utilizing molecular docking has emerged as a useful method for structure-based drug discovery [52]. Researchers have understood the activity of small molecules at binding sites on target proteins and revealed fundamental biochemical mechanisms through molecular docking models which illustrate the interaction between a ligand and a protein. Docking studies can facilitate the swift identification of a potential inhibitor for the target protein. 

SHBG is a glycoprotein produced by the liver that regulates the levels of sex hormones in the blood by binding to them [53]. Its function is crucial for controlling the bioavailability of testosterone, a hormone essential for male reproductive health [54]. Genetic mutations in SHBG, particularly at the rs6259 and rs727428 loci, have been linked to male infertility [1,55]. These mutations can alter the binding capacity of SHBG, leading to reduced testosterone bioavailability and resulting in infertility, prostate cancer, or gonadal dysfunction [56]. ADAM17, also known as TACE, is a membrane-bound protein responsible for shedding various cell surface proteins, impacting cell signaling pathways related to inflammation and apoptosis [57,58]. This process is crucial for maintaining normal testicular function, as ADAM17 is critical in inducing apoptosis in germ cells. This mechanism is especially significant in the context of testicular torsion, a common urological condition among young males that disrupts blood flow to the testes, leading to tissue damage [59]. Inhibition of ADAM17 has been proposed as a therapeutic strategy to mitigate the impact of testicular torsion. DNase I is a protein involved in DNA fragmentation during apoptosis, a process critical for regulated cell death [60]. However, excessive DNase I activity can lead to abnormal sperm DNA fragmentation, a factor in male infertility [61]. Clinical studies have indicated that inhibiting DNase I could reduce sperm DNA fragmentation, thereby offering a potential treatment approach for certain types of male infertility [62,63].

The docking results revealed substantial binding affinities between acanthoic acid and SHBG, ADAM17, and DNase I proteins, with binding affinities of −10.2, −6.8, and −5.8 kcal/mol, respectively, suggesting potential interactions influencing male fertility processes. For instance, acanthoic acid forms hydrogen bonds with specific amino acid residues in the N-terminal G domain of SHBG, such as Asp65, Asn82, and Lys134. In ADAM17, hydrophobic interactions occur with critical residues in the protein active site, including Leu348, Val402, His405, and Ala439. Regarding DNase I, acanthoic acid interacts with two histidine residues, His134 and His252, through π–alkyl interactions and forms hydrogen bonds with acidic residue Glu78, as well as Arg41 and Tyr76, in the protein active site.

The utilization of MD simulations offers a comprehensive understanding of the structural stability and dynamic behavior of acanthoic acid when interacting with SHBG, ADAM17, and DNase I proteins. Analysis of RMSD and RMSF elucidates the stability of these complexes throughout the 100 ns MD trajectory, with fluctuations observed in specific residues that do not impact the overall binding stability. The consistent presence of hydrogen bonds throughout the simulation underscores the enduring stability of the interactions between acanthoic acid and the target proteins. Additionally, the MM-GBSA analysis provides further insights into the energetic components driving the binding interactions, identifying key amino acid residues crucial for ligand binding within the binding sites of the target proteins.

In conclusion, this study demonstrates the promising potential of acanthoic acid to improve egg-hatching rates in *Drosophila melanogaster* under thermal stress, highlighting its possible role as an ingredient in dietary supplements or functional foods to enhance reproductive success, including addressing male infertility, in adverse environmental conditions. Based on these results, further studies could explore potential therapeutic applications of acanthoic acid in treating male infertility, including safety, pharmacokinetics, and efficacy evaluations, in animal models and clinical trials. Additionally, the impact of acanthoic acid on female fertility and overall reproductive health will be considered for a comprehensive understanding of its broader implications.

## Figures and Tables

**Table 1:** Effect of acanthoic acid on *Drosophila* egg-hatching rates.

Repeat	25 °C Controls		28 °C Controls		DMSO (28 °C)		Acanthoic Acid (28 °C)			
							1 μM		10 μM	
	Eggs	Hatched	Eggs	Hatched	Eggs	Hatched	Eggs	Hatched	Eggs	Hatched
1	73	57	81	0	17	0	32	6	130	8
2	47	47	28	0	68	3	44	17	63	17
3	18	16	59	0	18	1	46	15	102	74
4	26	18	15	0	51	0	36	8	37	17
5	15	10	15	0	43	6	15	7	20	9

**Table 2:** Binding affinity and binding interactions of acanthoic acid with proteins related to male infertility.

Target Protein	Binding Energy (kcal/mol)	Hydrogen Bonds	van der Waals Interactions	Hydrophobic Interactions
SHBG	−10.2	Asp65 Asn82 Lys134	Thr40, Ser41, Ser42, Phe56, Gly58, Asp59, Thr60, Trp66, Trp84, Lys106, Ser128, Leu131, Ile141	Leu80 (alkyl) Val112 (alkyl) Met139 (alkyl) Leu171 (alkyl) Phe67 (π–alkyl) Val105 (π–alkyl) Met107 (π–alkyl)
ADAM17	−6.8		Thr347, Gly349, Glu406, Tyr436, Pro437, Ile438	Leu348 (alkyl) Val402 (alkyl) Ala439 (alkyl) His405 (π–σ) His415 (π–alkyl)
DNase I	−5.8	Arg41 Tyr76 Glu78 Arg111	Glu39, Asn170	Pro137 (alkyl) His134 (π–alkyl) Tyr175 (π–alkyl) Tyr211 (π–alkyl) His252 (π–alkyl)

**Table 3:** Comparison of docking and MM-GBSA energies for acanthoic acid binding to SHBG, ADAM17, and DNase I.

Complex	Binding Energy (kcal/mol)	MM-GBSA Energy (kcal/mol)
SHBG–acanthoic acid	−10.2	−27.65
ADAM17–acanthoic acid	−6.8	−16.48
DNase I–acanthoic acid	−5.8	−6.37

## Data Availability

Data that support the findings of this study are available in the article.

## Supplementary Materials

The following supporting information can be downloaded at: https://www.mdpi.com/article/10.3390/cimb46070440/s1, Figure S1: Radius of gyration plots for the molecular dynamics simulations of the binding of acanthoic acid with SHBG, ADAM17, and DNase I; Figure S2: DSSP plots for the secondary structure transitions in the complexes of acanthoic acid with SHBG, ADAM17, and DNase I during 100 ns molecular dynamics simulations; Table S1: Main interactions of acanthoic acid with target proteins identified by the Protein–Ligand Interaction Profiler (PLIP) web tool.
