# Supplementary material for: Assessing the Efficacy of Acanthoic Acid Isolated from Acanthopanax koreanum Nakai in Male Infertility: An In Vivo and In Silico Approach
Source: Curr Issues Mol Biol. 2024 Jul 13;46(7):7411–29. doi: 10.3390/cimb46070440 (PMC11276288; doi:10.3390/cimb46070440)
Supplement: Supplementary file 1 [file cimb-46-00440-s001.zip › cimb-3082317-supplementary.pdf]

SUPPLEMENTARY MATERIAL

Assessing the Efficacy of Acanthoic Acid Isolated from *Acanthopanax koreanum* Nakai in Male Infertility: An In Vivo and In Silico Approach

Nguyen Viet Phong [1,†], Hyo-Sung Kim [2,3,4,†], Hyun-Jung Park [5], Eunbyul Yeom [2,3,4,*] and Seo Young Yang [1,*]

1 Department of Biology Education, Teachers College and Institute for Phylogenomics and Evolution, Kyungpook National University, Daegu 41566, Republic of Korea; ngvietphong@gmail.com
2 School of Life Science and Biotechnology, College of Natural Sciences, Kyungpook National University, Daegu 41566, Republic of Korea; gytjd98@gmail.com
3 School of Life Sciences, BK21 FOUR KNU Creative BioResearch Group, Kyungpook National University, Daegu 41566, Republic of Korea
4 KNU-G LAMP Project Group, KNU-Institute of Basic Sciences, School of Life Sciences, College of Natural Sciences, Kyungpook National University, Daegu 41566, Republic of Korea
5 Department of Plant Life and Resource Science, Sangji University, Wonju-si 26339, Republic of Korea; parkhj02@sangji.ac.kr
* Correspondence: yeb@knu.ac.kr (E.Y.); syy@knu.ac.kr (S.Y.Y.); Tel.: +82-53-950-5387 (E.Y.); +82-53-950-5910 (S.Y.Y.)
† These authors contributed equally to this work.

Table S1. Main interactions of acanthoic acid with target proteins identified by the Protein–Ligand Interaction Profiler (PLIP) web tool.

Target protein	Hydrophobic interactions (Distance, Å)	Hydrogen bonds (Distance D-A, Å)	Salt bridges (Distance, Å)
**SHBG**	Phe67 (3.39, 3.53)	Asp65 (3.02, 147.71°)	Lys134 (2.71)
	Val105 (3.12)	Asn82 (2.80, 159.37°)	
	Val112 (3.61)		
	Leu131 (3.93)		
	Leu171 (3.48)		
**ADAM17**	Thr347 (3.78)		
	Leu348 (3.41)		
	Val402 (3.84)		
	Ala439 (3.95)		
**DNase I**	Pro137 (3.46)	Tyr76 (3.80, 145.66°)	Arg41 (4.70)
	Tyr175 (3.81)	Tyr78 (3.95, 143.95°)	Arg111 (5.07)

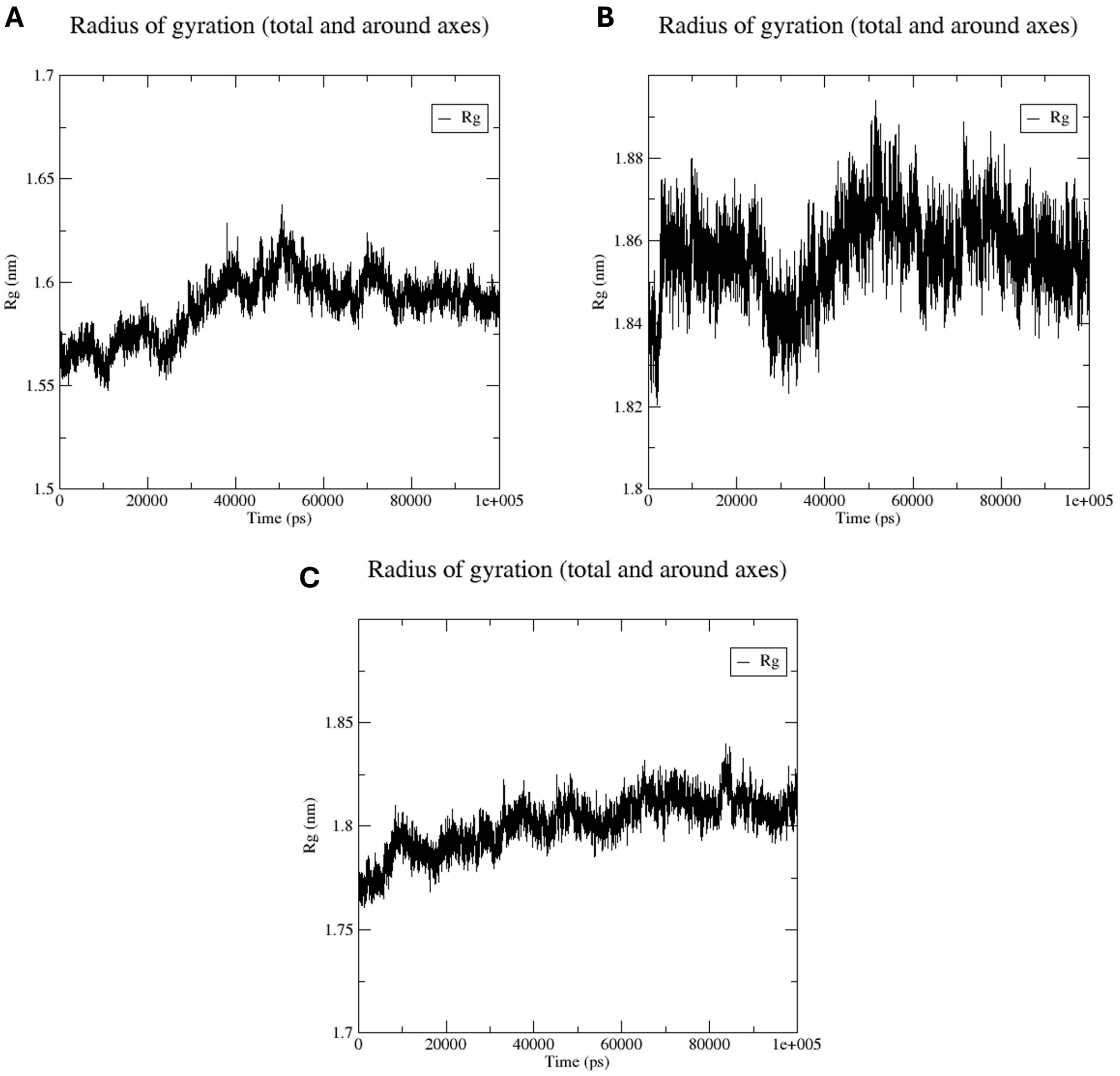

Figure S1. Radius of gyration plots for the molecular dynamics simulations of the binding of acanthoic acid with SHBG (**A**), ADAM17 (**B**), and DNase I (**C**).

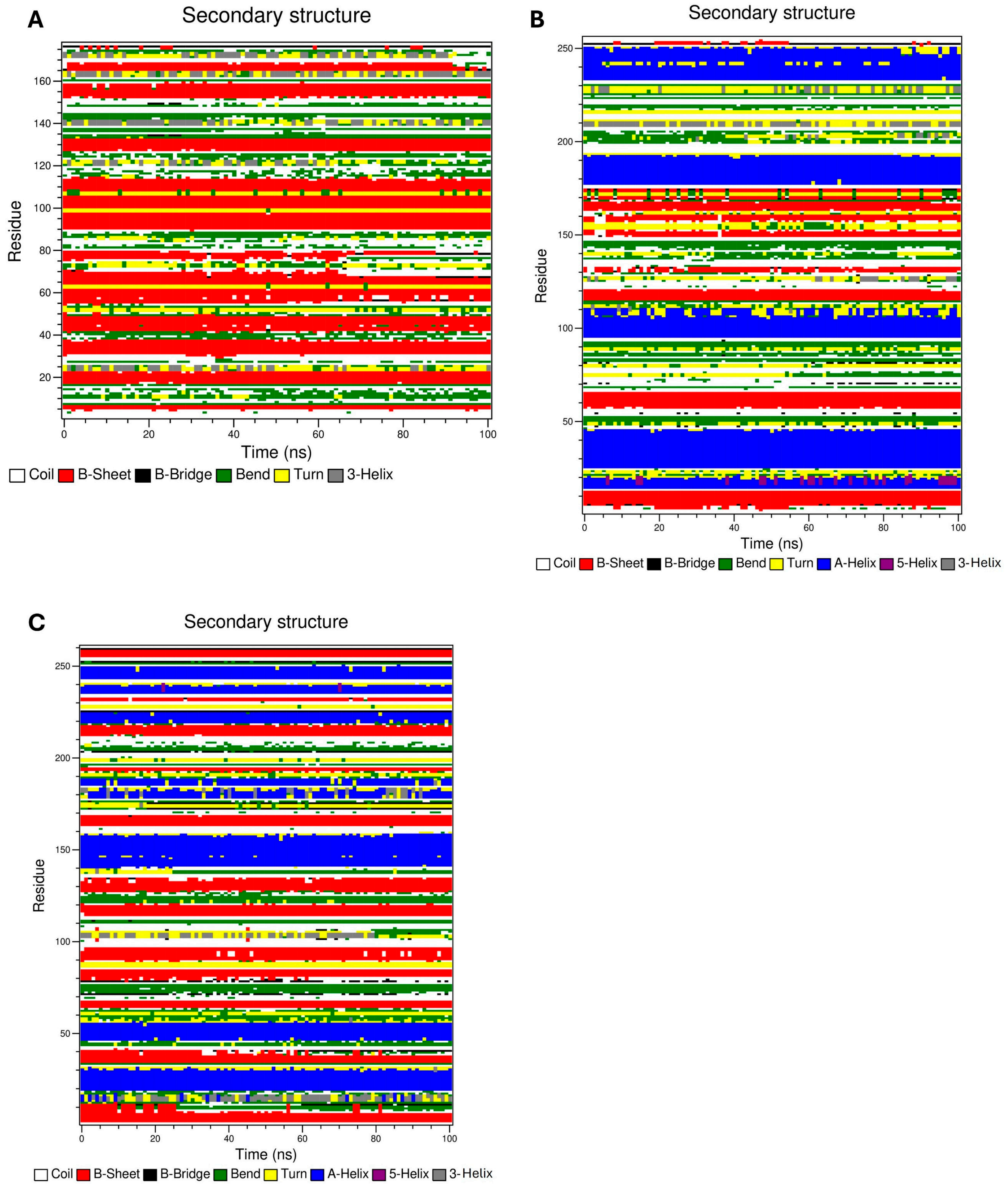

Figure S2. DSSP plots for the secondary structure transitions in the complexes of acanthoic acid with SHBG (**A**), ADAM17 (**B**), and DNase I (**C**) during 100 ns molecular dynamics simulations.
